# Application of Er,Cr:YSGG laser versus photopolymerization after silver diamine fluoride in primary teeth

**DOI:** 10.1038/s41598-021-00204-x

**Published:** 2021-10-21

**Authors:** Mohamed Hassan, Eman Bakhurji, Rasha AlSheikh

**Affiliations:** 1 Department of Preventive Dental Services, College of Dentistry, King Faisal University, Al-Hasa, Saudi Arabia; 2 Department of Preventative Dental Sciences, College of Dentistry, Imam Abdulrahman Bin Faisal University, Dammam, Saudi Arabia; 3 Department of Restorative Dental Sciences, College of Dentistry, Imam Abdulrahman Bin Faisal University, Dammam, Saudi Arabia

**Keywords:** Dental biomaterials, Paediatric dentistry

## Abstract

Examine the effect of dental curing light and laser treatments applied after Silver Diamine Fluoride (SDF) on dentin hardness in carious primary molars. This in-vitro study consisted of 30 extracted primary molars with caries extending into dentin without pulpal involvement. The collected teeth were randomly divided into three groups: group 1: received SDF then Sub-ablative low-energy of Er,Cr:YSGG laser, group 2: received SDF followed by application of curing light for 40 s, group 3: had SDF treatment only. In all groups, 38% Ag (NH3)2F SDF was used. Vickers hardness test was performed on sound dentin below carious lesion. Kruskal–Wallis Test was used to determine the mean difference in dentin hardness of the groups at 5% Significance level using SPSS software. Surface hardness of sound dentin below the carious lesion was statistically significantly higher in the laser + SDF group (891.24 ± 37.33 kgf/mm^2^) versus the two other groups (Light cure + SDF = 266.65 ± 90.81 kgf/mm^2^ and SDF only = 117.91 ± 19.19 kgf/mm^2^) with *p*-value ≤ 0.001. Although Photopolymerization of SDF increases the surface hardness of sound dentin below the carious lesion, applying laser after SDF has the highest surface hardness due to the laser’s sub-ablation of dentin.

## Introduction

SDF is an off-label FDA-approved agent to arrest active carious lesions and prevent their progression^1^. It is recommended to arrest cavitated carious lesions in children and adolescents, including those with special healthcare needs^2,3^. Recent studies stated that SDF is effective in arresting caries in permeant teeth too as well as root caries in the elderly^4^. SDF has several mechanisms of action; anti-microbial action by inhibiting biofilm formation and matrix metalloproteinase activities, and remineralization of demineralized tooth structure reducing loss of calcium and phosphate ions as well as collagen damage^1,5–10^. SDF has different features that are beneficial in clinical dentistry; control of caries, simplicity of use, affordability, minimal training needed, and being minimally-invasive^6^. Several clinical trials have reported SDF efficacy in managing caries in young children and primary teeth^11–16^.

Laser use is becoming more prominent in dental care with many lasers available in the dental market to perform different tasks depending on their wavelength. The Argon (514 nm) and diode lasers (800–980 nm) are primarily soft tissue lasers with affinity to pigmented tissues such hemoglobin and melanin. The Nd:YAG laser at a wavelength of 1064 nm has a high affinity to melanin but less affinity to hemoglobin when compared to the argon laser. The CO_2_ laser at a wavelength of 10,600 nm it has a high affinity to water and is second to the erbium family of lasers. It has been reported that laser can increase fluoride uptake by enamel, dentine, and root caries^17^. Zhao et al. reported improved enamel demineralization and acid resistance when SDF was applied after the use of a 9.3 µm carbon dioxide laser. The authors attributed the increased resistance to intrinsic changes in the enamel structure caused by the carbon dioxide laser irradiation followed by SDF application. They further reported less bacterial adhesion to the laser treated enamel^18^. Luk et al. reported similar findings when use carbon dioxide laser followed by SDF application^19^.

It has also been stated that enamel and dentine become more resistant to caries when combining fluoride with laser irradiation than when using any separately. The application of SDF followed by Er:YAG laser irradiation had the potential to melt and seal the dentinal tubules which prevented the further penetration of SDF deeper into dentin. Also, the sealing effect of the dentinal tubules decreased the sensitivity of the tooth^17,20–22^. While using the Er:YAG laser in sub-ablative energy after SDF application, Mei et al. reported increased microhardness, modulus of elasticity and fluoride uptake when compared to laser alone and SDF alone^23^. The Er,Cr:YSGG (erbium, chromium, yttrium, scandium, gallium garnet) is a hydrokinetic laser in which water is energized by the emitted photons leading to molecular excitation and localized micro-expansions by which the tooth surface is cut. The power output can vary between 0 and 6 W. The Er,Cr:YSGG laser uses a pulsed beam system, fiber delivery and a sapphire or glass tips bathed in a mixture of air and water vapor. The Er,Cr:YSGG laser at a wave length of 2.79 µm has the highest affinity to water and is reported to be more effective in cutting tooth structure since it is highly absorbed in both water and hydroxyapatite. The CO_2_, Er:YAG and the Er:Cr:YSGG lasers have a high affinity to be absorbed by water and hydroxyapatite, when comparaing four different lasers, Mei et al. reported higher fluoride uptake in the CO_2_ and Er:YAG SDF treated dentin groups when compared to the diode and Nd:YAG laser groups^24^.

On the other hand, the light cure has the potential to increase the hardness of dentin following the SDF application. Fung et al. reported higher caries arresting rates in anterior teeth compared to posterior ones after the application of SDF^25^. Crystal et al. believed that this could be because anterior teeth are more exposed to natural light compared to posterior ones which can cause more precipitation of silver ions in less amount of soaking time^26^. Another ex-vivo study investigating the effect of light cure following SDF application showed that the addition of light cure after SDF application have significantly increased dentin hardness around the carious lesion and had less penetration of SDF into dentin^27^. It is hypnotized that: (1) The addition of laser and light cure treatments following SDF application will increase the dentin hardness around the carious lesion compared to SDF alone. (2) Dentin hardness of the Laser treated group will be different from that of the light-cured group.

## Methods

This study was an in-vitro laboratory study to determine the effect of laser treatment versus light cure applied to SDF-treated teeth on dentin hardness. All methods were carried out in accordance with relevant guidelines and regulations related to experiments using extracted human teeth. Informed consents were obtained from patients for the use of their extracted teeth in the study. Ethical approval for this research was obtained from the Research Unit at the College of Dentistry, Imam Abdulrahman Bin Faisal University. The experiment consisted of three study groups using extracted primary molars with caries extending into dentin but without pulpal involvement. The teeth were selected based on the following criteria: 1. Extracted due to extensive dental caries extending into dentin. 2. Show no clinical signs of developmental anomalies. 3. Show no signs of pulpal exposure due to caries. The collected teeth were randomly divided into three groups: group 1: SDF and laser treatment, group 2: SDF and light cure, group 3: SDF only.

The sample size was estimated based on a prior study comparing dentin hardness after SDF alone and SDF followed by light cure application^27^. The mean dentin hardness of the SDF alone group = 558.07 kgf/mm^2^ and the mean hardness of the SDF with light cure group = 702.26 kgf/mm^2^, the standard deviation of the test group = 144.6 kgf/mm^2^, power of 80% and significance level of 5%, with an estimated size of effect of 1.5. Based on that, the estimated sample size would be 9 teeth per group, a total of 27 teeth (https://www.statskingdom.com/sample_size_all.html). The sample size was increased by 5% to compensate for laboratory errors. Therefore, the final sample size is 30 teeth (10 teeth/group).

All the surrounding soft tissues after extraction were removed and teeth were cleaned from blood and stored in sterile saline (saline is changed every 2 days to prevent bacterial growth). SDF (38% Ag (NH3)2F) (Advantage Arrest, 2018 Elevate Oral Care, LLC., West Palm Beach, FL) was applied on all the teeth of all groups after drying the tooth for 30 s using a disposable micro-brush. During the first round of application, group 1: received SDF then Sub-ablative low-energy of Er,Cr:YSGG laser pulses contious p (Waterlase MD, Biolase Technology Inc., CA, USA), irradiated for 10 s in scan motion mode, at 1 mm irradiance distance utilizing a G4 MZ6 tip (6 mm length) with a diameter of 600 µm in pulsed wave mode. The power output was with 0.5 W, 5 Hz, without water cooling, and 55% of air^28,29^. Group 2: received SDF followed by application of curing light (LEDition, Ivoclar Vivadent AG, Schaan/Liechtenstein, USA) for 40 s, group 3 received SDF only. After 2 weeks, the three groups had the same applications again. The teeth were stored in sterile saline in an incubator at 37 °C after each application and whenever the teeth were not in use. After the second application, the teeth were sectioned vertically using the (IsoMet 5000 Linear Precision Saw, Buehler, Illinois, USA).

**Figure 1:**
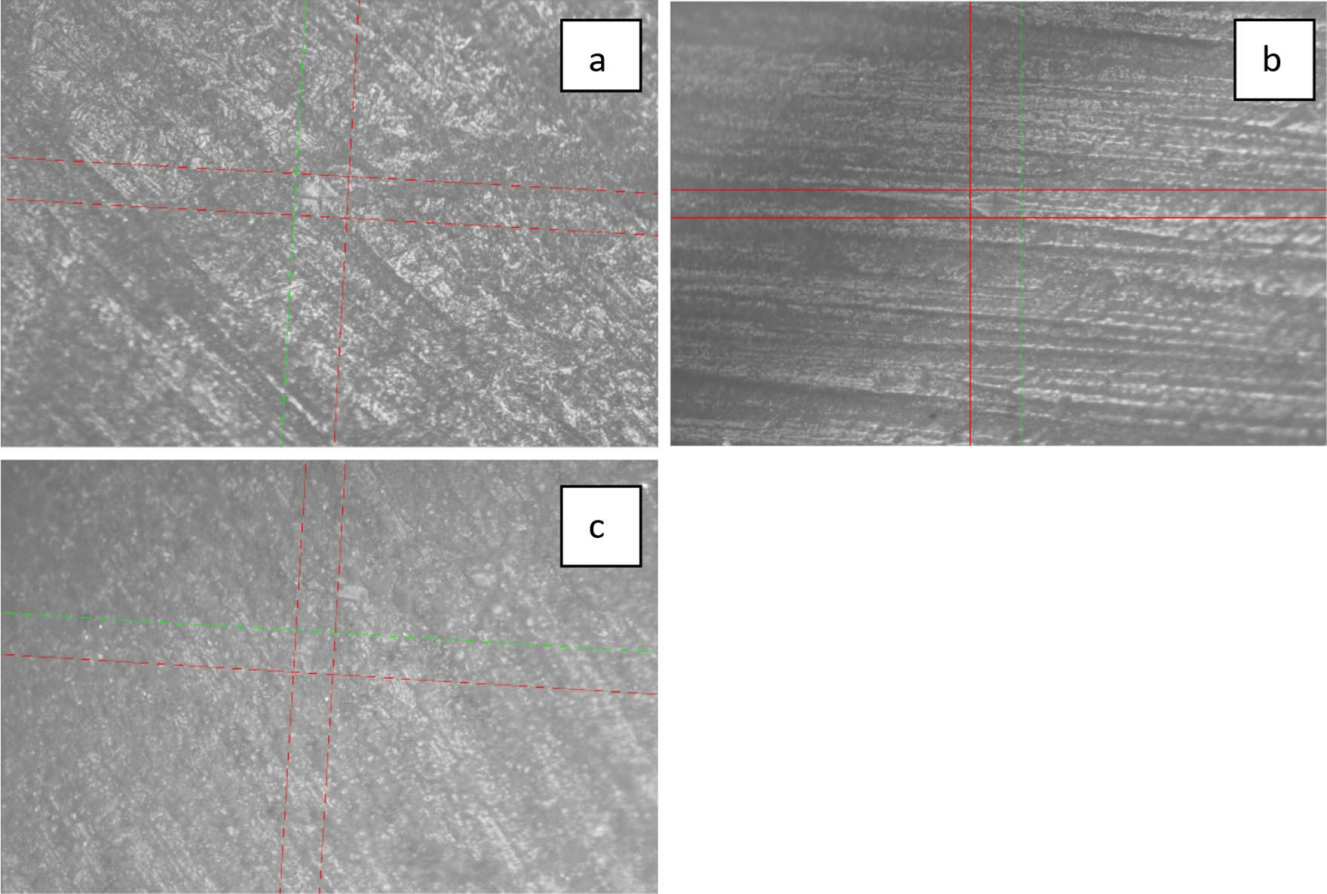
Samples of Pyramidal Indentation for Vickers Surface Hardness Test of Sound Dentin below the Carious Lesion in the Three Study Groups [(**a**) Laser + SDF group, (**b**) Light cure + SDF, (**c**) SDF alone].

After sectioning the teeth, the hardness of sound dentin below the infected dentin was evaluated using micro-hardness values of the dentine slice surfaces which was assessed by a pyramidal diamond tip with a diameter of 20 nm secured to a nano-indenter at room temperature (MicroMet 6040 Microhardness Testing Machine, Buehler, Illinois, USA) as shown in Fig. 1. The force for the indentation was 100 mN, and 3 indentations per sample were performed randomly on each specimen with a 2 mm distance from each other. Vickers hardness test of the specimens was calculated, and the load–displacement curve was recorded for the 3 readings in each sample. Then the average of the 3 readings was calculated.

### Statistical analysis

Kruskal–Wallis Test was used to test the mean difference in dentin hardness of the groups at 5% Significance level using SPSSSoftware Program Version 9.3, Second Edition. Post hoc test was used to identify differences within the groups.

## Results

**Figure 2:**
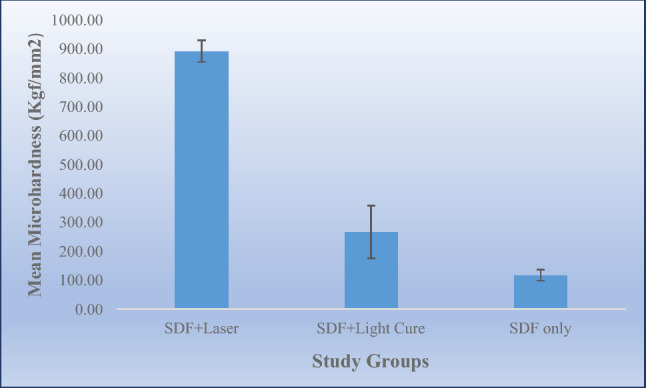
Mean ± SD of hardness (kgf/mm^2^) in sound dentin below the carious lesion in the study groups.

Kruskal–Wallis Test showed the mean surface hardness of sound dentin below the carious lesion was statistically significantly higher in the laser + SDF group (mean ± SD = 891.24 ± 37.33 kgf/mm^2^) versus the two other groups (Light cure + SDF mean ± SD = 266.65 ± 90.81 kgf/mm^2^ and SDF only mean ± SD = 117.91 ± 19.19 kgf/mm^2^) with *p*-value ≤ 0.001 (Fig. 2).

The post-hoc test showed that all three groups were statistically significantly different from each other (*p*-value ≤ 0.001) as shown in Table 1. The mean difference was the highest between the laser + SDF and the SDF groups (773.33 kgf/mm^2^) while the lowest mean difference of dentine hardness was between the light cure + SDF group and the SDF group (148.74 kgf/mm^2^).

**Table 1:** Post hoc test for microhardness mean difference of the three study groups.

Study groups	Mean difference of microhardness (kgf/mm^2^)	Standard error	*p*-value
Laser + SDF Vs SDF alone	773.33	25.83	≤ 0.001
Light cure + SDF Vs SDF alone	148.74	25.83	≤ 0.001
Laser + SDF Vs Light cure + SDF	624.59	25.83	≤ 0.001

## Discussion

The action of SDF on carious lesion is still under investigation, it is widely believed that the antibacterial action of silver helps arrest the further progression of the carious lesion while the fluoride helps to remineralize the tooth structure. The purpose of this in-vitro study was to investigate the effect of the Er,Cr:YSGG laser and light-curing on the dentin microhardness following SDF application. The use of the Er,Cr:YSGG laser after SDF application provided significantly higher micro-hardness values when compared to the microhardness measurement of the light cure + SDF and the SDF groups.

The findings in the laser + SDF group can be attributed to different mechanisms of action. First, silver reacts with oxygen, phosphorus, and sulfur present in the caries lesion leading to the formation of silver phosphate, silver oxide, and silver sulfide^30^. Silver sulfide is a dense black solid that is insoluble which may explain the increased surface hardness after SDF application^31^.

Dental lasers are known to cause structural changes to enamel and dentin. Yu, et al. under field emission scanning electron microscope (FE-SEM), reported deep holes of more than 1 mm depth when the Er,Cr:YSGG laser was used to prepare the dentinal surface with a power output of 5 watts, 20 Hz for 5 s. Most of the dentinal tubules were open^32^. Mozammal, et al. using the Er,Cr:YSGG laser at 3 watts power, 70% air, and 20% water observed the dentinal surface under the scanning electron microscope. The walls and bottoms of the cavity were smooth. No smear layer was seen, and the dentinal tubules were clearly visible. When the laser was used without water, the heating of dentin and later cooling created a molten lava-like appearance with micro-holes could be seen^33^.

Secondly, various studies have shown increased uptake of fluoride and improved acid resistance when lasers were used in combination with fluoride application. Hicks, et al. observed a significant reduction in caries progression when the Argon laser was used alone or in conjunction with APF gel compared to the control sample^34^. Westerman et al. showed a 50% reduction in lesion depth when argon lasers were used in combination with acidulated phosphate fluoride gel (APF) when compared to no treatment control. Argon laser alone caused a 20% reduction in the carious lesion depth when compared to the no treatment control^35^. Using the Er:YAG (2940 nm) laser and similar parameters as the present study, Mei et al. achieved the highest fluoride uptake when compared to the CO_2_ laser, Nd:YAG laser and the Diode laser^24^. Both the Er:YAG (2940 nm) and the Er,Cr:YSGG (2780 nm) have the highest absorption to water and hydroxyapatite leading to significant changes when used in ablative and sub-ablative energy levels.

A third possible mechanism of action is the structural changes caused by lasers. Dental lasers are known to cause structural changes to enamel and dentin. When used with sub-ablative low energy levels without water, thermal changes are seen on dentinal surfaces leading to the formation of molten lava-like appearance with micro-holes and fissures, these thermal changes have been hypothesized to increase fluoride intake^33^. Furthermore, lasers have been shown to alter the microstructure of radicular dentin in sub-ablative levels^36^. The increase presence of cracks and surface roughness may play an important role in the increased fluoride uptake^24^. Lasers have been shown to alter the Ca and P weight percentage present in dentin^23^. The structural changes caused by lasers on dentinal surfaces are believed to have a synergistic effect leading to increased surfaces hardness and acid resistance^37^. Whether these structural changes are advantageous or not needs to be further investigated^38^.

Hajizadeh et al. showed increased microhardness in demineralized dentin when the Cr,Er:YSGG laser was used in combination with a fluoridated agent. Surprisingly, when the laser was used alone, a decrease in microhardness of dentin was noted^38^. Mei et al. studied SDF application in dentin after pre-treatment with an Er:YAG laser, they found the laser + SDF group to have a significantly higher microhardness measurement when compared to all other groups. Furthermore, the fluoride content was significantly higher in the laser + SDF group when compared to the SDF alone group, the laser alone group, and the water-only “control group”. The sliver (Ag) content was similar in both of the laser + SDF group and the SDF alone group^30^.

In the current study, the SDF + light cure group did show a significant difference in the microhardness measurements when compared to the SDF alone. Similar findings were reported by Toopchi et al., who attributed the increased hardness to the light reduction of SDF and the formation of silver sulfide, a hard, insoluble dense black structure. Higher content of silver deposition in dentin was also reported by the Toopchi et al. study. The authors attributed the increased silver surface deposition to the light induced reduction of SDF which limits the penetration depth and maintain most of the silver at the surface^27^.

Microhardness has been used in research as an indirect measurement of mineral content in the tooth structure. Dentin hardness is a well-accepted indirect measurement of mineral content in dentin, remineralization, and cessation of further carious lesion progression. The increased uptake of fluoride in the laser-treated teeth may explain the increased microhardness in the laser + SDF group in our study.

The shortcomings of the in-vitro studies testing microhardness are the inability to truly measure the microhardness in an environment like that of the oral cavity. Sample dehydration influences the hardness test, and therefore, different protocols such as the use of ethanol have been suggested to rehydrate the samples. The comparison of hardness test results among different studies is another shortcoming of in vitro studies, sample preparation techniques may yield different hardness results. Similarly, the difference in the amount and method of the load application can change the hardness test measurement outcome. Lastly, the lesion depth and location on the indentations of the hardness test can greatly influence the hardness test measurement^39^.

In the current study, a significant difference was found among all three groups which help support our conclusion. Furthermore, the standardized measurements in our study yield the comparison between the three study groups applicable and valid. Further clinical studies are needed to confirm the findings of the current study and its clinical application.

In conclusion, although Photopolymerization of SDF increases the surface hardness of sound dentin below the carious lesion, applying laser after SDF has the highest surface hardness due to their different mechanism of action. The laser causes sub-ablation of dentin while the light cure reacts with the silver particles making the dentin harder.
